# Differential effects of different delivery methods on progression to severe postpartum hemorrhage between Chinese nulliparous and multiparous women: a retrospective cohort study

**DOI:** 10.1186/s12884-020-03351-7

**Published:** 2020-10-31

**Authors:** Chang Xu, Wanting Zhong, Qiang Fu, Li Yi, Yuqing Deng, Zhaohui Cheng, Xiaojun Lin, Miao Cai, Shilin Zhong, Manli Wang, Hongbing Tao, Haoling Xiong, Xin Jiang, Yun Chen

**Affiliations:** 1 Department of medical administration, Beijing University Shenzhen Hospital, Shenzhen, 518036 China; 2 Department of medical administration, Zhuhai People’s Hospital (Zhuhai hospital affiliated with Jinan University), Zhuhai, 519000 China; 3 Department of Epidemiology and Biostatistics, College for Public Health and Social Justice, Saint Louis University, St. Louis, MO 63013 USA; 4 Department of Health Statistics and Research Development, Chongqing Health Information Center, Chongqing, 401120 China; 5 West China School of Public Health and West China Fourth Hospital, Sichuan University, Chengdu, 610041 China; 6 China Center for Special Economic Zone Research, Shenzhen University, Shenzhen, 518060 Guangdong China; 7 School of Medicine and Health Management, Tongji Medical College, Huazhong University of Science and Technology, Wuhan, 430016 China

**Keywords:** Delivery methods, Multiparous, Nulliparous, Postpartum hemorrhage

## Abstract

**Background:**

Delivery methods are associated with postpartum hemorrhage (PPH) both in nulliparous and multiparous women. However, few studies have examined the difference in this association between nulliparous and multiparous women. This study aimed to explore the difference of maternal and neonatal characteristics and delivery methods between Chinese nulliparous and multiparous women, and then examine the differential effects of different delivery methods on PPH between these two-type women.

**Methods:**

Totally 151,333 medical records of women who gave birth between April 2013 to May 2016 were obtained from the electronic health records (EHR) in a northern province, China. The severity of PPH was estimated and classified into blood loss at the level of < 900 ml, 900–1500 ml, 1500–2100 ml, and > 2100 ml. Neonatal and maternal characteristics related to PPH were derived from the same database. Multiple ordinal logistic regression was used to estimate associations.

**Results:**

Medical comorbidities, placenta previa and accreta were higher in the nulliparous group and the episiotomy rate was higher in the multiparous group. Compared with spontaneous vaginal delivery (SVD), the adjusted odds (aOR) for progression to severe PPH due to the forceps-assisted delivery was much higher in multiparous women (aOR: 9.32; 95% CI: 3.66–23.71) than in nulliparous women (aOR: 1.70; 95% CI: 0.91–3.18). The (aOR) for progression to severe PPH due to cesarean section (CS) compared to SVD was twice as high in the multiparous women (aOR: 4.32; 95% CI: 3.03–6.14) as in the nulliparous women (aOR: 2.04; 95% CI: 1.40–2.97). However, the (aOR) for progression to severe PPH due to episiotomy compared to SVD between multiparous (aOR: 1.24; 95% CI: 0.96–1.62) and nulliparous women (aOR: 1.55; 95% CI: 0.92–2.60) was not significantly different. The (aOR) for progression to severe PPH due to vacuum-assisted delivery compared to SVD in multiparous women (aOR: 2.41; 95% CI: 0.36–16.29) was not significantly different from the nulliparous women (aOR: 1.05; 95% CI: 0.40–2.73).

**Conclusions:**

Forceps-assisted delivery and CS methods were found to increase the risk of severity of the PPH. The adverse effects were even greater for multiparous women. Episiotomy and the vacuum-assisted delivery, and SVD were similar to the risk of progression to severe PPH in either nulliparous or multiparous women. Our findings have implications for the obstetric decision on the choice of delivery methods, maternal and neonatal health care, and obstetric quality control.

**Supplementary Information:**

**Supplementary information** accompanies this paper at 10.1186/s12884-020-03351-7.

## Background

Postpartum hemorrhage (PPH) is a common and fatal postpartum complication in parturient women [1]. Globally, 22% of maternal mortality is directly caused by PPH, although mortality rates among regions vary significantly [2]. A recent study reported that the PPH-related death in Africa was 34%; Asia, 31%; Latin American, 21%; and developed countries, 13% [3]. In China, PPH is by far the leading cause of maternal death (32%) [4].

Exploring the risk factors for PPH contributes to the clinical work and maternal and neonatal health. The risk factors associated with PPH have been identified in previous studies [5–7], which can be divided into four categories: maternal, neonatal, fetal appendage, and delivery methods [6, 8]. Among all risk factors, delivery methods play an important role [9]. Cesarean section (CS) has the greatest risk for severe PPH and leads to worse outcomes, such as hysterectomy [7, 10]. Episiotomy has been found to be associated with PPH [11–13]. A few studies suggested that an assisted method of birth is a protective factor against PPH, while others showed no significant difference in PPH between assisted-delivery methods and spontaneous vaginal delivery (SVD) [14, 15].

Most PPH-related studies about the difference between nulliparous and multiparous women are generally consistent in that compared with nulliparous, being multiparous would be a protective factor of PPH [6, 8, 13, 15–19]. The reason behide this is perhaps that previous studies just analyzed their samples without considering the differential effects of delivery methods on the specific parturients that might be modified by parity, i.e. nulliparous or multiparous. Furthermore, risk factors for PPH were not equally studied between nulliparous and multiparous. There are many more studies that focused on nulliparous pregnancy and low-risk pregnancy than multiparous pregnancy [20–22]. The reason behind that might be the clinical characteristics of the nulliparous are more accessible than those of the multiparous, especially in random control trials (RCTs) [23–29]. Previous studies have shown that no matter in the nulliparous samples or in the multiparous samples, different delivery methods are associated with PPH [6, 8, 14]. Those studies don’t compare PPH due to delivery methods between nulliparous and multiparous women. No study was found to examine how parity modifies the association between PPH and delivery methods. Moreover, studies on the risk factors associated with PPH using a large sample size are also scarce.

To fulfill the gaps, this study was designed to take advantage of a large Chinese sample and identify the difference in the severity of PPH caused by various delivery methods between nulliparous and multiparous. The hypothesis of this study includes:1) Chinese nulliparous and multiparous women present statistical difference among maternal characteristics, neonatal characteristics, and the delivery methods;2) After xcluding the confounding factors, there are statistical differences in PPH of nulliparous and multiparous women under different delivery methods.

## Methods

### Study design

This study will determine the sample size according to certain inclusion and exclusion criteria. Then, according to the purpose and hypothesis of this research,this study firstly analyzed the difference of maternal characteristics, neonatal characteristics, and the delivery methods between the nulliparous and multiparous women. On the basis of the basic situation, this study then examined the associations between maternal, neonatal characteristics, delivery methods and the severity of PPH in the nulliparous and multiparous women, respectively. After that, this study will use regression analysis methods to explore the effects of different delivery methods on PPH of the nulliparous and multiparous women, on the basis of comprehensive consideration of maternal characteristics and neonatal characteristics.

### Sample

The retrospective sample cohort was extracted from the databank of a standardized administrative system of electronic health records (EHRs) maintained by the Health Commission in Shanxi province of China. The databank has more than 220 variables that describe medical characteristics for each parturient, including basic demographic information, essential diagnosis (up to 10), pathological diagnosis, surgical procedure (up to 7), categories of cost and subcategories of service charges, length of stay, outcomes at discharge, etc. All diagnoses and surgical procedures at birth were coded based on the International Classification of Diseases code (ICD-10-CM) and procedure code (ICD-9-CM3). The rigorous process for the data collection used by the databank was described as followed: a chief physician in a hospital reviewed every case in the hospital initially. Then, trained coders certified by the Medical Record Management Association (MRMA) entered the patient’s data into the administrative system and submitted it to a case manager of the hospital for crosscheck before uploading the data to the databank to ensure the accuracy and quality.

**Fig. 1:**
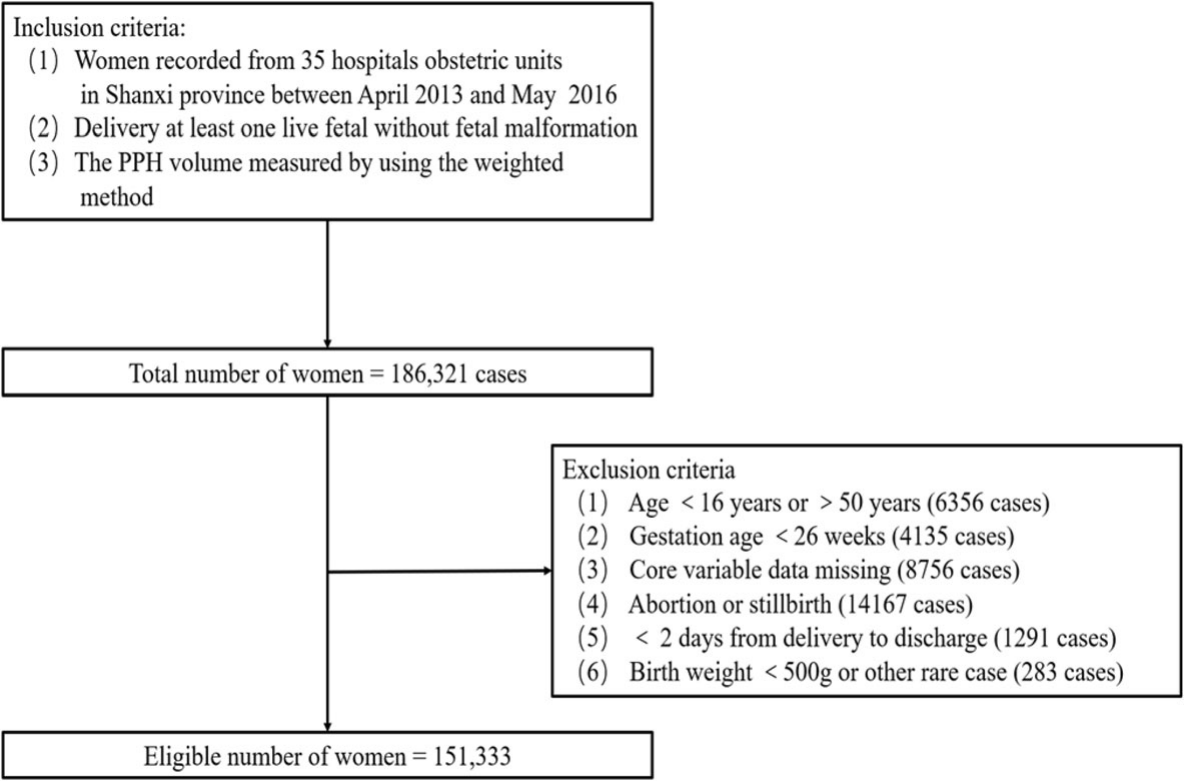
Flow chart of the sampling process (the inclusion and exclusion criteria)

In this research, parturient women who had a history of prior gestations lasting > 20 weeks that ended in abortion or other pregnancy termination methods were classified as multiparous. Women whose gestations lasted < 20 weeks or had no previous pregnancies were classified as the nulliparous [8]. According to the definition of research sample, the selection of the sample for this study was carried out based on the following inclusion and exclusion criteria as shown in Fig. 1, and the relative explanation are as follows:

The inclusion criteria included: (1) women recorded from 35 hospitals obstetric units; (2) delivery at least on live fetal without fetal malformation; (3) the PPH volume measured by using the weighted method. The exclusion criteria included: (1) Age < 16 years or > 50 years; (2) gestation age < 26 weeks; (3) Core variable data missing; (4) abortion or stillbirth; (5) < 2 days from delivery to discharge; (6) birth weight < 500 g or other rare case.

The explanation needed is as below:

- This study explores PPH differences between nulliparous and multiparous women based on different delivery methods. If the fetus is known to be malformed, or the miscarriage or stillbirth needs to be carried out, some women’s delivery methods will be affected as a result. At the same time, the purpose or focus of these maternal deliveries will change from paying attention to the common safety of the maternal and neonatal to choosing a delivery method that protects the maternal as much as possible to reduce maternal trauma (such as adopting a destructive delivery method after fetal death). This change in the purpose of childbirth will lead to heterogeneity in the research object. Therefore, to avoid discussing this situation and reducing the heterogeneity of the research object, this study specifically excluded fetal malformations, miscarriages, and stillbirths, so that only women who successfully completed the delivery process were taken as the research object.
- The relevant diagnosis of this study mainly comes from the medical record data. The Chinese Medical Records Office stipulates that patient information should be archived and the relevant audits can start only 3 days after admission. In this case, the medical records of women with “≤ 2 days from delivery to discharge” will face missing information. Therefore, to ensure quality, we removed maternal information of the sample objects with “≤ 2 days from delivery to discharge” as missing data.
- Another reason for “≤ 2 days from delivery to discharge” may be local customs. Our research sample area is affected by traditional customs, so some women may require to be discharged within 2 days of admission or even the admission day after delivery (< 24 h). This caused the weighing method could not be used in this sample to collect their corresponding PPH, so this part of the data was excluded.
- In addition to the weighing method mentioned in this research, judging PPH in this study has an auxiliary method, which is to roughly judge the difference between the hemoglobin immediately after admission and the third day after delivery. This auxiliary judgment method is to judge whether the bleeding of the weighing method is realistic. In our database, most of the pregnant women who are “less than 2 days from delivery to discharge” have not tested the postpartum hemoglobin, and there are obvious information deficiencies in the estimation and verification of bleeding. In order to keep the rigor and reliability of the sample, we eliminated the women with “<2 days from delivery to discharge”.

### The postpartum hemorrhage

The maternal hemorrhage was defined as the volume of hemorrhage within 24 h of delivery. There were several methods available to estimate the volume of PPH [30–32]. Most obstetricians used the visual method of approximation, which usually had a 30–50% deviation [30, 31]. To improve the estimation accuracy, a weighing method for hemorrhage evaluation during the 24 h postpartum period was used [16, 33]. It followed a process: a weighed cotton pad was placed under the parturient’s perineum before the labor. Then, the blood-infiltrated pad was reweighed to calculate the difference. Other bleeding supplies utilized including gauze, bandage, and other pieces of cotton were also weighed and recorded. Subtracting the amniotic fluid volume (AFV) from the summed total weight of 24-h PPH was divided by the specific gravity of the blood (1.05) to obtain PPH volume. The AFV was determined by the Cavalieri method on ultrasound images at the inspection [34] and obtained from the supplement data of the EHRs.

The volume of PPH was divided into four categories according to the obstetrical hemorrhage grading standard recommended by Benndetti [35]. Specifically, the blood loss volume < 900 ml (i.e. < 15% of total blood volume, TBV) was marked as Level 0 (L0), considering as no PPH; the blood loss volume between 900 and 1499 ml (i.e. 15–25% of TBV) was labeled as Level 1 (L1), meaning mild PPH; the blood loss volume from 1500 to 2099 ml (i.e. 25–35% of TBV) was as labeled as Level 2 (L2), meaning moderate PPH; and the blood loss volume > 2100 ml (i.e. > 35% of TBV) was as Level 3 (L3), meaning severe PPH. This kind of PPH categorization is a reference to the advanced trauma life support (ATLS) classification, whose aim is to discuss the situation of severe PPH in detail, including the vital signs of parturients. Moreover, it is of help for clinical application in obstetrical operation. The severity of PPH was treated as an ordinal variable based on the categorical volume of PPH.

### Delivery methods

In this research, the primary independent variable was delivery methods that were classified as CS, vaginal delivery (VD)- episiotomy, VD- forceps-assisted delivery, VD- vacuum-assisted delivery, and SVD without instrument suggested by Liu et al. [36] In the clinical practice, an episiotomy is not a strict method of delivery. However, as a commonly used delivery method or preoperative operation, an episiotomy is closely related to the delivery method. Some literature reported that episiotomy is an independent risk factor for PPH [37]. Therefore, this study will include episiotomy into the delivery method, which is more conducive to observing the effect of this method on PPH in Chinese nulliparous and multiparous women. Dummy variables were created for these methods with SVD used as the reference category. Due to the small number of parturient women who underwent both episiotomy and forceps-assisted, or both episiotomy and vacuum-assisted delivery, those patients were combined into the forceps-assisted or vacuum-assisted group, respectively. Parity was classified as nulliparous and multiparous women.

### Maternal characteristics

Maternal age was classified into five groups: 16–19 years, 20–24 years, 25–29 years, 30–34 years, and older than 34 years. Pregnant women with 25–29 years were the reference group. The women’s condition at admission was classified into: normal, emergency and serious. The complications of pregnancy were recorded as to whether uterine inertia, soft birth canal tumor, and/or preeclampsia occurred. Chronic diseases of the women included whether women had cardiovascular diseases, respiratory diseases, hepatopathy, nephropathy, venereal disease, rhesus hemolytic disease, or coagulopathy during the gestational period. The definitions of the maternal variables were exhibited in “Supplementary for maternal characteristics definition in Table 1”.

### Neonatal characteristics

Gestational age was classified into 26–36 weeks, 37–39 weeks, 40–42 weeks, and above 42 weeks. The 40–42 weeks group was the reference group. Neonatal birth weight was classified into 500–2499, 2500-3999, and 4000 g or more. Amniotic fluid volume abnormality was classified into none, polyhydramnios, and oligohydramnios. Other neonatal characteristics included vertex malposition, twins or multiplets pregnancy, placenta previa, placenta accrete, placental abruption, placental retention, and premature rupture of membrane (PROM). The definitions of neonatal variables were exhibited in “Supplementary for neonatal characteristics definition in Table 2”.

### Statistical analysis

Chi-square test was conducted to compare maternal characteristics, neonatal characteristics, and the delivery methods between the nulliparous and multiparous women [8]. Chi-square test was also used to examine associations between maternal, neonatal characteristics, delivery methods and the severity of PPH in the nulliparous and multiparous women, respectively. Chi-square test was also applied to screen the finally incorporates maternal and neonatal variables into a multiple ordinal logistic regression model.

Multiple ordinal logistic regression was used to regress the severity of PPH on the delivery methods and other risk factors since the severity of PPH was an ordinal variable with four levels. Cumulative logits were estimated across four levels of PPH. Whether the proportional odds assumption of the cumulative logits was met was checked using the score chi-square test. Since patients were clustered in different hospitals, the assumption of independence of observations for any statistical test was not met in this sample. Therefore, the robust variance estimation was used in conjunction with ordinal logistic regression to account for the non-independence of patients [36]. The variance inflation factor was utilized to check the collinearity between variables. A stepwise method was used to determine the significance of independent variables in the multiple ordinal logistic regression model. The final model was built on the significant variables. Odds ratios (OR) and 95% confidence intervals (CI) were reported to quantify the associations. An estimated OR from the multiple ordinal logistic regression model was interpreted as the effect of an independent variable on the odds of having a more severe level of PPH compared to a less severe level of PPH level. This OR for an independent variable was the same no matter what cutoff point that separated PPH into a more severe and a less severe level. This interpretation implied that similar conclusions were reached when the different cutoff point of the PPH severity was used in studying a predictor’s effect. All analyses were performed using Stata version 14.0 (Statistics/Data Analysis, College Station, TX, USA) [38]. All tests were two-tailed with a significance level of .05.

## Results

There were 112, 907 (74.61%) nulliparous women and 38, 426 (25.39%) multiparous women in the sample. The maternal characteristics stratified by parity are shown in Table 1. The normal admission rate was higher in the multiparous group than that in the nulliparous group, while the emergency admission rate was higher in the nulliparous group. There were more women with 30 years or older in the multiparous group than those in the nulliparous group. The prevalence of woman’s obstetric complication and medical comorbidity was higher in the nulliparous than the multiparous group.

**Table 1:** The distribution of maternal characteristics stratified by parity

Variables	Nulliparous	Multiparous	*p*-value^a^
	*N* = 112,907(%)	*N* = 38,426(%)	
**Maternal characteristics**			
Age (years)			<0.01
16–19	1727(1.53)#	452(1.18)	
20–24	22,828 (20.22)	6707 (17.45)	
25-29^b^	52,846 (46.80)	16,824 (43.78)	
30–34	23,992 (21.25)	9733 (25.33)	
> 34	11,514 (10.20)	4710 (12.26)	
Admission			<0.01
Normal^b^	100,793 (89.27)	36,201 (94.21)	
Emergency	4500(3.99)	179(0.47)	
Serious	7614(6.74)	2046(5.32)	
Uterine inertia^c^	3639(3.22)	466(1.21)	<0.01
Soft birth canal tumour	4002(3.54)	559(1.45)	<0.01
Preeclampsia	6551(5.80)	890(2.32)	<0.01
Cardiovascular diseases	7007(6.21)	1011(2.63)	<0.01
Respiratory disease	374(0.33)	44(0.11)	<0.01
Hepatopathy	2989(2.65)	548(1.43)	<0.01
Nephropathy	641(0.57)	49(0.13)	<0.01
Venereal disease	356(0.32)	65(0.17)	<0.01
Rhesus hemolytic disease	277(0.25)	53(0.14)	<0.01
Coagulopathy	64(0.06)	4(0.01)	<0.01

Note: ^#^The percentage is the proportion of the row total, not column total. The percentages are compared vertically, not horizontally. ^a^ *p*-values were derived from the chi-square test; ^b^ Reference group; ^c^ Including psychological and anesthetic factors, meanwhile excluded the uterine inertia cases that were caused by other factors mentioned in the same model

Table 2 shows neonatal characteristics and delivery methods in the two groups. The prevalences of the youngest and the oldest gestational age were higher in the nulliparous group. The prevalence of infants with low birth weight and overweight were twice as high in the nulliparous as those in the multiparous group. The prevalences of placenta previa, placenta accreta and placental abruption, oligohydramnios, and PROM were higher in the nulliparous group. The prevalence of cesarean section and forceps-assisted method were higher in the nulliparous group, while the prevalence of episiotomy was much higher in the multiparous group. The SVD prevalence was about the same in two groups.

**Table 2:** The distribution of neonatal characteristics and delivery methods stratified by parity

Variables	Nulliparous	Multiparous	*p*-value^a^
	*N* = 112,907(%)	*N* = 38,426(%)	
**Neonatal characteristics**			
Gestation age			<0.01
26-36w	17,513 (15.51)#	3556(9.25)	
37-39w	43,734 (38.73)	14,740 (38.36)	
40-42w^b^	50,526 (44.75)	19,880 (51.74)	
Above 42w	1134(1.00)	250(0.65)	
Neonatal birth weight			<0.01
500-2499 g	11,613 (10.29)	2159(5.62)	
2500–3999g^b^	89,868 (79.59)	33,970 (88.40)	
above 4000 g	11,426 (10.12)	2297(5.98)	
Vertex malposition	3007(2.66)	98(0.26)	<0.01
Twins or multiplets pregnancy	3285(2.91)	871(2.27)	<0.01
Placenta previa	1833(1.62)	295(0.77)	<0.01
Placenta accreta	92(0.08)	10(0.03)	<0.01
Placental abruption	1167(1.03)	167(0.43)	<0.01
Placental retention	541(0.48)	115(0.30)	<0.01
Amniotic fluid volume abnormality			<0.01
None^b^	101,805 (90.17)	36,315 (94.51)	
Polyhydramnios	716(0.63)	219(0.57)	
Oligohydramnios	10,386(9.20)	1892(4.92)	
PROM	20,413 (18.08)	3895 (10.14)	<0.01
**Delivery methods**			<0.01
Cesarean section	50,543 (44.77)	104,44 (27.18)	
VD- Episiotomy	12,961 (11.48)	10,184 (26.50)	
VD- Forceps-assisted	650(0.58)	107(0.28)	
VD- Vacuum-assisted	230(0.20)	56(0.15)	
SVD without instruments^b^	48,523 (42.98)	17,635 (45.89)	

Note: ^#^The percentage is the proportion of the row total. The percentages are compared vertically, not horizontally. *PROM* Premature rupture of membrane, *SVD* Spontaneous vaginal delivery

^a^*, p-*value derived from the chi-square test; ^b^, Reference group

The distributions of delivery methods and other covariates stratified by parity and PPH severity are displayed in Table 3. In the nulliparous group, the prevalence of PPH associated with episiotomy at L0 and L1 were approximately the same. In the multiparous group, the prevalence of PPH associated with episiotomy at L0 was twice as likely as that at L1. The prevalence of PPH associated with episiotomy at L2 and L3, respectively, was higher in the multiparous than that in the nulliparous group. The prevalence of PPH associated with the forceps-assisted method was approximately identical at L0 and L1, while the prevalence of PPH associated with the forceps-assisted method was greater at L1 than that at L0.

**Table 3:** The distribution of maternal and neonatal characteristics by different levels of postpartum hemorrhage in the sample stratified by parity

Variables	Nulliparous				*p* -values^a^	Multiparous				*p*-value^a^
	<900 ml	900-1499 ml	1500-2100 ml	>2100 ml		<900 ml	900-1499 ml	1500-2100 ml	>2100 ml	
	L0	L1	L2	L3		L0	L1	L2	L3	
	*N* = 108,606(%)	*N* = 3638(%)	*N* = 480(%)	*N* = 183(%)		*N* = 37,689(%)	*N* = 635(%)	*N* = 75(%)	*N* = 27(%)	
Delivery method					<0.01					<0.01
Cesarean section	47,957 (44.16)#	2095 (57.59)	359 (74.80)	132 (72.13)		9947 (26.39)	423 (66.62)	55 (73.33)	19 (70.37)	
VD- Episiotomy	12,440 (11.45)	493 (13.55)	23(4.79)	5(2.73)		10,091 (26.77)	84 (13.23)	7(9.33)	2(7.41)	
VD- Forceps-assisted	622(0.57)	22(0.60)	5(1.04)	1(0.55)		98(0.26)	8(1.26)	0(0.00)	1(3.70)	
VD- Vacuum-assisted	223(0.21)	7(0.19)	0(0.00)	0(0.00)		55(0.15)	1(0.16)	0(0.00)	0(0.00)	
SVD without instruments^b^	47,364 (43.61)	1021 (28.06)	93 (19.38)	45 (24.59)		17,498 (46.43)	119 (18.74)	13 (17.33)	5 (18.52)	
Placenta previa	1515(1.39)	243(6.68)	55 (11.46)	20 (10.93)	<0.01	219(0.58)	53(8.35)	14 (18.67)	9 (33.33)	<0.01
Placenta accreta	54(0.05)	23(0.63)	6(1.25)	9(4.92)	<0.01	2(0.01)	2(0.31)	3(4.00)	3 (11.11)	<0.01
Placental abruption	997(0.92)	132(3.63)	26(5.42)	12(6.56)	<0.01	145(0.38)	16(2.52)	3(4.00)	3 (11.11)	<0.01
Placental retention	453(0.42)	60(1.65)	17(3.54)	11(6.01)	<0.01	90(0.24)	17(2.68)	6(8.00)	2(7.41)	<0.01
Uterine inertia ^c^	3318(3.06)	257(7.06)	41(8.54)	23 (12.57)	<0.01	414(1.10)	41(6.46)	6(8.00)	5 (18.52)	<0.01
Vertex malposition	2891(2.66)	101(2.78)	11(2.29)	4(2.19)	0.9	91(0.24)	7(1.10)	0(0.00)	0(0.00)	<0.01
Respiratory disease	325(0.30)	33(0.91)	7(1.46)	9(4.92)	<0.01	39(0.10)	4(0.63)	0(0.00)	1(3.70)	<0.01
Hepatopathy	2303(2.12)	649 (17.84)	19(3.96)	16(9.84)	<0.01	527(1.40)	19(2.99)	1(1.33)	1(3.70)	<0.01
Nephropathy	575(0.53)	45(1.24)	9(1.88)	12(6.56)	<0.01	44 (0.12)	2(0.31)	0(0.00)	3 (11.11)	<0.01
Rhesus hemolytic disease	231(0.21)	40(1.10)	6(1.25)	0(0.00)	<0.01	47(0.12)	5 (0.79)	1(1.33)	0(0.00)	<0.01
Severe coagulopathy	24(0.02)	14(0.38)	8(1.67)	18(9.84)	<0.01	2(0.01)	0(0.00)	0(0.00)	2(7.41)	<0.01
Preeclampsia	5992(5.52)	448 (12.32)	83 (17.29)	28 (15.30)	<0.01	818(2.17)	60(9.45)	9 (12.00)	3 (11.11)	<0.01

Note: ^#^The percentage is the proportion of the row total. The percentages are compared vertically, not horizontally. SVD, spontaneous vaginal delivery. ^a^ *p*-value derived from the chi-square test; ^b^ Reference group. ^c^ Including psychological and anesthetic factors and excluding the uterine inertia cases that cause by the other factors mentioned in our model already

The prevalence of PPH associated with SVD was greatest at L0, followed by L1, L3, and L2 in the nulliparous group, while the prevalence of PPH associated with SVD was greatest at L0, followed by L2, L1 and L3 in the multiparous group. Also, the disorders of the placenta were associated with a higher volume of PPH. Although this upward trend was present in both groups, it was even more severe in the multiparous group. The prevalence of PPH associated with respiratory disease, nephropathy and severe coagulopathy increased from L0 to L3 in both groups. The prevalence of PPH associated with rhesus hemolytic disease was the greatest at L2 in both groups. The prevalence of PPH associated with preeclampsia was the greatest at L2 in the nulliparous group, but approximately identical at L2 and L3 in the multiparous group.

**Fig. 2:**
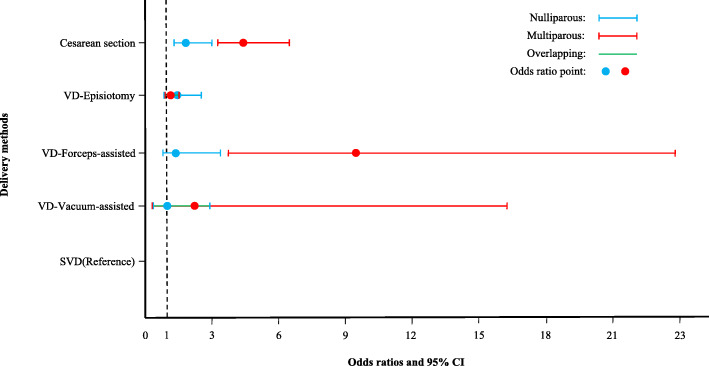
The effects of delivery methods on postpartum hemorrhage in nulliparous and multiparous groups. The blue line: Nulliparous. The red line: Multiparous. The green line: Overlapping. The dotted line: The value of the aOR equals to 1. The point: The value of the aOR

The adjusted odds ratios (aORs) and 95% CIs derived from the final multiple ordinal logistic regression model for significant variables are presented in Table 4. In comparison with the odds of SVD, the odds of increased PPH volume for CS in the multiparous women (aOR: 4.32; 95% CI: 3.03–6.14) was more than twice that in the nulliparous women (aOR: 2.04; 95% CI: 1.40–2.97). The 95% CIs were not overlapping (Fig. 2). However, PPH severity for episiotomy was not significantly higher than the SVD in both nulliparous women (aOR: 1.55; 95% CI: 0.92–2.60) and multiparous (aOR: 1.24; 95% CI: 0.96–1.62). The risk for a greater volume of PPH in the forceps-assisted delivery group was much higher in the multiparous women (aOR: 9.32; 95% CI: 3.66–23.71) than in the nulliparous women (aOR: 1.70; 95% CI: 0.91–3.18). The 95% CIs were separated (Fig. 2). Meanwhile, the risk for greater volume of PPH in the vacuum-assisted delivery group was neither significant in the multiparous women (aOR: 2.41; 95% CI: 0.36–16.29) nor the nulliparous women (aOR: 1.05; 95% CI: 0.40–2.73).

**Table 4:** Adjusted odds ratio and 95% confidence intervals for all independent variables from ordinal logistic regression analysis of postpartum hemorrhage

Variables	Nulliparous	Multiparous
**Core differentiated variables**		
Delivery method		
Cesarean section	2.04 (1.40–2.97)	4.32 (3.03–6.14)
VD- Episiotomy	1.55 (0.92–2.60)	1.24 (0.96–1.62)
VD- Forceps-assisted	1.70 (0.91–3.18)	9.32 (3.66–23.71)
VD- Vacuum-assisted	1.05 (0.40–2.73)	2.41 (0.36–16.29)
SVD without any instruments	Reference	
Placenta previa	4.97 (3.50–7.07)	8.64 (5.84–12.80)
Placenta accreta	10.45 (5.77–18.90)	70.21 (1.20–411.96)
Placental abruption	3.19 (2.26–4.50)	4.25 (2.57–7.01)
Placental retention	5.35 (3.47–8.24)	8.16 (4.80–13.89)
Uterine inertia	3.03 (1.48–6.21)	5.54 (1.76–17.50)
Vertex malposition	0.87 (0.63–1.22)	1.93 (1.25–3.00)
Respiratory disease	2.63 (1.75–3.94)	3.26 (1.53–6.93)
Hepatopathy	8.92 (2.31–34.48)	1.51 (0.80–2.86)
Nephropathy	1.90 (1.22–2.93)	5.55 (1.63–18.88)
Rhesus hemolytic disease	7.52 (3.11–18.17)	6.44 (2.94–14.08)
Coagulopathy	47.41 (23.32–96.37)	111.37 (2.27–546.2)
**Other confounders**		
Age (years)		
16–19	1.32 (0.92–1.89)	2.38 (1.17–4.84)
20–24	1.24 (1.11–1.39)	1.32 (1.00–1.74)
25–29	Reference	
30–34	0.98 (0.88–1.09)	1.25 (0.98–1.59)
> 34	1.23 (1.05–1.44)	1.36 (1.09–1.68)
Admission		
Normal	Reference	
Emergency	2.73 (0.96–7.76)	2.49 (1.12–5.54)
Serious	1.60 (0.98–2.60)	1.71 (1.19–2.46)
Soft birth canal disorder	0.89 (0.65–1.20)	1.26 (0.84–1.89)
Cardio-cerebrovascular diseases	1.15 (0.90–1.46)	1.24 (0.91–1.67)
Venereal disease	3.05 (0.94–9.89)	2.42 (0.71–8.16)
Gestation age (week)		
26–36	1.20 (0.95–1.50)	1.16 (0.84–1.60)
37–39	0.90 (0.55–1.47)	1.09 (0.86–1.39)
40–42	Reference	
> 42	1.56 (1.33–1.92)	1.48 (0.76–2.89)
Neonatal birth weight (g)		
500–2499	0.83 (0.66–1.05)	0.99 (0.71–1.38)
2500–3999	Reference	
> 4000 g	1.57 (1.31–1.72)	1.68 (1.23–1.96)
Twins or multiplets pregnancy	2.24 (1.65–3.05)	2.02 (1.05–3.88)
Amniotic fluid volume abnormality		
None	Reference	
Polyhydramnios	1.15 (0.80–1.67)	1.65 (0.78–3.49)
Oligohydramnios	0.75 (0.60–0.92)	0.83 (0.61–1.12)
PROM	0.74 (0.60–0.92)	1.02 (0.75–1.40)

Note: *SVD* Spontaneous vaginal delivery, *PROM* Premature rupture of membrane

In Table 4, the factors relating to the placenta were noted to be of strong influence on PPH severity. Those adverse effects were much stronger in the multiparous women than nulliparous women. Specifically, a greater effect of placenta previa on PPH severity was stronger in the multiparous women (aOR: 8.64; 95% CI: 5.84–12.80) than that in the nulliparous women (aOR: 4.97; 95% CI: 3.50–7.07). Similarly, a greater effect of placental retention on PPH severity was stronger in the multiparous women (aOR: 8.16; 95% CI: 4.80–13.89) than that in the nulliparous women (aOR: 5.35; 95% CI: 3.47–8.24). A slightly larger effect of placental abruption on PPH severity was observed in the multiparous women (aOR: 4.25; 95% CI: 2.57–7.01) than that in the nulliparous women (aOR: 3.19; 95% CI: 2.26–4.50). In contrast, an extremely greater effect of placenta accrete on PPH severity was observed in the multiparous women (aOR: 70.45; 95% CI: 1.20–411.96) than that in the multiparous women (aOR: 10.45; 95% CI: 5.77–18.90). The unusually high OR in the multiparous women suggests an imprecise estimate due to the spare data.

Uterine inertia was more prone to cause PPH in the multiparous women (aOR: 5.54; 95% CI: 1.76–17.50) than in the nulliparous women (aOR: 3.03; 95% CI: 1.48–6.21). In contrast to uterine inertia, vertex malposition presented a significant effect on PPH severity in the multiparous (aOR: 1.93; 95% CI: 1.25–3.00), but no significant effect in the nulliparous women (aOR: 0.87; 95% CI: 0.63–1.22). Preeclampsia had a similar effect on PPH severity in the multiparous (aOR: 2.09; 95% CI: 1.73–2.53) and nulliparous women (aOR: 1.78; 95% CI: 1.40–2.26). The association between respiratory diseases and PPH severity in the multiparous women (aOR: 3.26; 95% CI: 1.53–6.93) was stronger than that in the nulliparous women (aOR: 2.63; 95% CI: 1.75–3.94). Nephropathy presented a considerably greater effect on PPH severity in the multiparous (aOR: 5.55; 95% CI: 1.63–18.88) than that in the nulliparous women (aOR: 1.90; 95% CI: 1.22–2.93). Despite the obvious risk of PPH associated with coagulopathy, the multiparous women (aOR: 111.37; 95% CI: 2.27–546.2) presented an extremely higher risk for PPH severity than that in the nulliparous (aOR: 47.41; 95% CI: 23.32–96.37).

However, hepatopathy presented a greater effect on PPH severity in the nulliparous (aOR: 8.92; 95% CI: 2.31–34.48) than that in the multiparous (aOR: 1.51; 95% CI: 0.80–2.86). Rhesus hemolytic disease also showed a greater effect on PPH severity in the nulliparous women (aOR: 7.52; 95% CI: 3.11–18.17) than that in the multiparous women (aOR: 6.44; 95% CI: 2.94–14.08). To highlight the effects of delivery methods on the severity of PPH, adjusted ORs and 95% CIs for four delivery methods compared to the SVD presented in Table 4 are shown in Fig. 2.

## Discussion

This research firstly compared the differences in maternal and neonatal characteristics between nulliparous and multiparous groups. As for maternal characteristics, the prevalence of obstetric complications and medical comorbidities in nulliparous women is higher than that in the multiparous group, while for the neonatal characteristics, the incidence of placenta previa and accreta is also higher in nulliparous. Those results are nearly strange because nulliparous are generally younger than multiparous in western countries. The possible reasons for this phenomenon are: 1) China promulgated the “second child” policy in 2015. Affected by the previous traditional and restrictive fertility policy, most women who had comorbidities and complications during previous delivery would no longer choose to give birth again. In contrast, those who were in good health and had fewer comorbidities or complications would follow the new policy to carry out another pregnancy, which may greatly reduce the incidence of comorbidity and placenta previa and accreta among the multiparous group. 2) Women with a history of pregnancy and delivery have accumulated experience in pregnancy and childbirth. When they are pregnant again, they will apply the maternal health knowledge and skills learned during the previous pregnancy and delivery to the second pregnancy, that is, paying more attention to nutrition, exercise, and physical and mental health. This can relatively reduce the incidence of comorbidities among multiparous. Our finding indicates that women should strengthen their knowledge reserves during pregnancy and delivery and emphasis the nutrition and physical and mental health during pregnancy, to reduce the occurrence of obstetric complications and medical comorbidities as much as possible.

In comparing the differences in maternal and neonatal characteristics between nulliparous and multiparous, this study also found that the rate of episiotomy was higher in the multiparous group. This may also be an odd phenomenon because the multiparous who generally experienced pregnancy and delivery will much easier and skilled when giving birth again. It can be seen from Table 2 that the number of low birth gestational age (26-36w) and low birth weight infants (500-2499 g) among nulliparous women is much larger than the number of that among multiparous. The parturient giving birth to the low weighted child does not need to adopt episiotomy for delivery. This dilutes the possibility of episiotomy in nulliparous under certain circumstances, which in turn reduces the probability of episiotomy in nulliparous. Of course, this is just a possible rational explanation. Another possibility is maybe the problematic nature and bias of the data in this study.

Then, the current study examined the severity of PPH in relation to five delivery methods in nulliparous and multiparous women, respectively, and compared the associations between these two groups. After controlling for a comprehensive list of neonatal conditions, maternal complications during pregnancy and women’s chronic diseases, we found that multiparous women who received CS or forceps-assisted delivery method had a much greater risk for progression to severe PPH than those who had SVD. This risk was also much higher for multiparous women than nulliparous women in China. Forceps-assisted delivery was not associated with a greater risk for PPH severity than SVD in nulliparous women. There is no difference in the risk for the severe PPH between episiotomy and SVD and between vacuum-assisted delivery and SVD in both nulliparous women and multiparous. The moderating effects of parity on different delivery methods with PPH severity are evident. In addition, the observation that the risk for progression to severe PPH in the multiparous women was higher than that in the nulliparous women is approximately consistent across most of the risk factors being considered.

Previous studies reported that the multiparous pregnancy had a higher risk for PPH than nulliparous pregnancy [6, 8, 13, 15, 16, 19, 39], when parity was included as one of the independent variables. This study shows that multiparous women have a greater risk for progression to severe PPH than nulliparous women if they received CS or forceps-assisted delivery. It is well known that CS can cause an increased rate of obstetric mortality and morbidity [38, 40, 41]. We did not find that the multiparous women had a significantly lower risk for progression to severe PPH than the nulliparous women if they received episiotomy or vacuum-assisted delivery. Our study is the first to find the moderating effect of parity on different delivery methods with PPH. Our findings are less likely to be an artifact due to four reasons. (1) The large sample size with 151,333 pregnant women being studied provides large statistical power to detect the adverse effect. This is especially true for the CS and episiotomy. Although the sample size is not very large for forceps-assisted delivery, it is comparable to many previous studies [6, 8, 40]. (2) This study included many more potential confounders for PPH than most previous similar studies [5, 6, 17, 39, 41–44] so that the greater risk for PPH in multiparous women was above and beyond other major risk factors leading to the severity of PPH. (3) The same regression model was applied in the nulliparous and multiparous group. (4) The size of OR for the CS and forceps-assisted delivery is not trivial, respectively.

Consistent with other studies on birth methods [9, 14, 45, 46], this study has found that CS is an independent risk factor for PPH after other covariates are adjusted. This was in contrast to Shmueli et al. reporting that episiotomy was an independent PPH risk factor for Israeli nulliparous women and multiparous women [37]. Furthermore, the results of our study show that among both nulliparous and multiparous women, episiotomy does not present a higher risk of PPH compared to SVD. The discrepancy may be due to differences in women’s factors for PPH and obstetrician’s experience.

The current literature indicates that assisted birth is a risk factor for PPH and the risk is much higher in multiparous women than in nulliparous women [16, 47, 48]. Consistently, the results of this study also found that the risk for progression to severe PPH from forceps-assisted birth was much higher than the SVD in multiparous, but not significantly in nulliparous. In addition, the prevalence of assisted birth in our sample is much lower than that reported in American and European studies [8, 49]. These discrepancies may be due to a much higher CS prevalence in China. In Chinese pregnant women with a low-risk pregnancy (i.e. vertex malposition or cord entanglement), a CS is more often used than assisted vaginal birth in Chinese OB/GYN clinics despite the absence of a complete trial of labor [43]. It means that the opportunities for assisted birth are significantly reduced in Chinese hospitals. Consequently, the concomitant decline of obstetrician’s competence in traditional assisted birth may have led to an increased vaginal-operative PPH prevalence in China.

This study has several strengths. It is the first to compare the association of progression to severe PPH with various delivery methods, adjusting for other maternal and neonatal risk factors for PPH between the nulliparous and multiparous women. The sample size was large enough to have greater precision in OR estimates for common and rare risk factors for PPH. In contrast to previous studies regarding PPH, an analytical strength of this study is to control the clustering effect of hospitals on patients using the robust variance estimation to provide unbiased parameter estimates. The usage of ordinal logistic regression takes advantage of the ordinality of PPH severity to gain greater power in statistical tests and no interval-scale assumption about distances between PPH severity levels [50]. The latter means that the results from this method do not depend much on the choice of the thresholds of PPH volume. This has an important implication for this study in that the estimated associations are not biased even though the volume of PPH was not measured as a continuous scale in milliliters. In addition, this analysis has included the most comprehensive covariates in the regression models and pregnant women at a different level of risk for PPH, compared to previous studies, randomized controlled trial (RCT) studies for the maternal and neonatal adverse outcomes that only included a limited number of confounding factors and some low-risk nulliparous women [17, 51]. Those studies did not have a direct comparison between the nulliparous and the multiparous pregnancies in regards to the association between PPH and delivery methods. They did not separate the SVD from assisted methods during VD (including the assisted delivery or other delivery with instruments).

### Limitations

These results should be interpreted cautiously. Firstly, one limitation is the weighting method of the blood loss. On one hand, the blood loss is not directly measured for its precise volume. Then type of PPH could not be established at the very least primary or secondary or both because Chinese medical records only record PPH amount without distinguishing its type. The accuracy of measuring PPH volumes in this duty is not perfect but better than that of the visual method. To account for this imprecise measure of the volume of blood loss, ordinal logistic regression is applied, in which PPH was an ordinal dependent variable. This method treats the real PPH volume as a latent variable with observed thresholds indicating volume levels. In addition, the statistical method is not sensitive to measurement variation that can result from other measurement methods used in collecting PPH volume. Therefore, the ORs are not biased. Secondly, another limitation is the accuracy of data, which may be questionable. The data time range of this study is from April 2013 to May 2016, which is relatively long from now. Then, according to the sample inclusion and exclusion criteria of this study, it may cause some valid data to be deleted to a certain extent. However, our research carried out the data analysis based on the large sample data, which can make up for the lack of data to a certain degree. Thirdly, it is also a limitation that the findings are obtained from a Chinese sample cohort in which the CS rate is higher than that in developed countries [52–54]. The moderating effect of parity may not hold in other countries. Further studies are needed to replicate this finding. Fourthly, the limitation is about parity history, namely, it is hard to accurately distinguish women who had birth 3 or more times. However, the proportion of those women with a high number of births in this sample should be very low due to the family planning policy in China. The last limitation is that these findings may not be generalizable to other racial or ethnic populations due to cultural and religious differences.

## Conclusion

Our findings have an implication for clinical practice and pregnant women. CS should be used with great caution in pregnant women, especially for multiparous women. To reduce PPH due to the forceps-assisted delivery method, there need to be modifications to the obstetrical forceps especially in China, namely changing the concept of Chinese obstetric management, paying more attention to instrument-assisted delivery, and increasing the proportion of midwives or strengthening training for the obstetrician midwifery. If needed, alternative assisted-methods such as episiotomy and vacuum-assisted methods are much safer to use.

## Supplementary Information

**Additional file 1.** Supplementary for Maternal Characteristics Definition in Table 1.

**Additional file 2.** Supplementary for Neonatal Characteristics Definition in Table 2.

**Additional file 3.** Description of the “Supplementary for maternal characteristics definition in Table 1” and Description of the “Supplementary for Neonatal Characteristics Definition in Table 2”.

## Data Availability

The datasets analyzed during the current study are not publicly available due to the Health Commision’s policy on patient safety and privacy but are available from the corresponding author on reasonable request.

## Abbreviations

PPH: Postpartum hemorrhage
CS: Cesarean section;
VD: Vaginal delivery
SVD: Spontaneous vaginal delivery;
PROM: Premature rupture of membrane;
OR: Odds ratio;
CI: Confidence interval
