# Correction to: Differential effects of different delivery methods on progression to severe postpartum hemorrhage between Chinese nulliparous and multiparous women: a retrospective cohort study

**DOI:** 10.1186/s12884-021-03560-8

**Published:** 2021-01-19

**Authors:** Chang Xu, Wanting Zhong, Qiang Fu, Li Yi, Yuqing Deng, Zhaohui Cheng, Xiaojun Lin, Miao Cai, Shilin Zhong, Manli Wang, Hongbing Tao, Haoling Xiong, Xin Jiang, Yun Chen

**Affiliations:** 1 Peking University Shenzhen Hospital, Shenzhen, 518036 China; 2 Department of Medical Administration, Zhuhai People’s Hospital (Zhuhai Hospital Affiliated with Jinan University), Zhuhai, 519000 China; 3 Department of Epidemiology and Biostatistics, College for Public Health and Social Justice, Saint Louis University, St. Louis, MO 63013 USA; 4 Department of Health Statistics and Research Development, Chongqing Health Information Center, Chongqing, 401120 China; 5 West China School of Public Health and West China Fourth Hospital, Sichuan University, Chengdu, 610041 China; 6 China Center for Special Economic Zone Research, Shenzhen University, Shenzhen, 518060 Guangdong China; 7 School of Medicine and Health Management, Tongji Medical College, Huazhong University of Science and Technology, Wuhan, 430016 China

**Correction to: BMC Pregnancy Childbirth 20, 660 (2020)**

**https://doi.org/10.1186/s12884-020-03351-7**

Following publication of the original article [1], the authors informed us that the first affiliation has been provided incorrectly. In this Correction, correct and incorrect version is shown.

Incorrect: “Department of medical administration, Beijing University Shenzhen Hospital, Shenzhen 518036, China.”

Correct: “Peking University Shenzhen Hospital, Shenzhen 518036, China.”

The original article has been corrected.
