# Supplementary material for: Differential effects of different delivery methods on progression to severe postpartum hemorrhage between Chinese nulliparous and multiparous women: a retrospective cohort study
Source: BMC Pregnancy Childbirth. 2020 Oct 31;20:660. doi: 10.1186/s12884-020-03351-7 (PMC7603680; doi:10.1186/s12884-020-03351-7)
Supplement: Supplementary file 1 — Additional file 1. Supplementary for Maternal Characteristics Definition in Table 1. [file 12884_2020_3351_MOESM1_ESM.docx]

**Description of the “Supplementary for maternal characteristics definition in Table 1”**

This supplementary file helps to explain the contents of Table 1 in the text of the manuscript. It exhibits the maternal characteristics variables’ definition and their origins. In this supplementary file, each obstetric definition of maternal characteristics is listed in the table in detail. These latest maternal concepts are from Chinese textbooks, which are nation-widely used in obstetrics practice in hospitals. Based on the standard definition, all statistical data related to obstetric diagnosis and treatment are collected. The purpose of this supplementary is to better enable our readers from different countries to understand the differences of the concepts and the details of obstetrics in China according to the standard. Additionally, it can make sure the readers understand the results of our research more objectively and comprehensively.

| Supplementary for Maternal Characteristics Definition in Table1 | | | |
| --- | --- | --- | --- |
| **Characteristics** | **Definition or Content** | **Origin** | **Year** |
| Uterine inertia | Uterine contraction function depends on the degree of synchronization of uterine myogenic, psychogenic and hormone regulation systems, and abnormalities in either side can lead to uterine atony. | Obstetrics and Gynecology-8th. People's Health Publishing House. Beijing, China | 2014 |
| Soft birth canal tumour | The soft birth canal consists of vagina, cervix, lower uterus and soft tissue of pelvic floor. The abnormalities of soft birth canal include vaginal septum, vaginal mediastinum, cervical adhesion, uterine fibroids and so on. | Obstetrics and Gynecology-8th. People's Health Publishing House. Beijing, China | 2014 |
| Preeclampsia | After 20 weeks of gestation, systolic blood pressure (> 140 mmHg) and/or diastolic blood pressure (> 90 mmHg) appeared, accompanied by urinary protein (> 0.3g/24h), or random urinary protein, or no proteinuria, but combined with any of the following: thrombocytopenia (< 100 *10 ^ 9/L); liver function damage; renal function damage; pulmonary edema; new central nervous system abnormalities or visual impairments. | Obstetrics and Gynecology-8th. People's Health Publishing House. Beijing, China | 2014 |
| Cardiovascular diseases | Cardiovascular diseases generally refer to ischemic or hemorrhagic diseases in the heart, brain and whole body caused by hyperlipidemia, blood viscosity, atherosclerosis, hypertension and so on. | Internal Medicine-7th. People's Health Publishing House. Beijing, China | 2012 |
| Respiratory disease | Respiratory disease is a common and frequently-occurring disease. The main pathological changes are trachea, bronchus, lungs and thoracic cavity. The mild ones suffer from coughing, chest pain and respiratory impairment. The severe ones suffer from dyspnea, hypoxia, and even respiratory failure, resulting in death. | Internal Medicine-7th. People's Health Publishing House. Beijing, China | 2012 |
| Hepatopathy | Hepatopathy is a disease that occurs in the liver. Including hepatitis B, hepatitis A, hepatitis C, cirrhosis, steatosis, liver cancer, alcoholic liver and many other liver diseases. | Internal Medicine-7th. People's Health Publishing House. Beijing, China | 2012 |
| Nephropathy | Nephropathy is a common disease that seriously endangers human health. It mainly includes different types of nephritis, acute renal failure, kidney stones, renal cysts and so on. | Internal Medicine-7th. People's Health Publishing House. Beijing, China | 2012 |
| Venereal disease | Various diseases transmitted through sexual contact, similar sexual behavior and indirect contact are collectively referred to as sexually transmitted diseases. The coverage of sexually transmitted diseases has been extended to cover at least 50 diseases caused by pathogenic microbial infections, including five traditional sexually transmitted diseases and non-gonococcal urethritis, condyloma acuminatum, genital herpes, AIDS, bacterial vaginosis, vulvovaginal candidiasis, vaginal trichomoniasis, scabies, vaginal lice and hepatitis B | Internal Medicine-7th. People's Health Publishing House. Beijing, China | 2012 |
| Rhesus hemolytic disease | Neonatal Rh hemolytic disease is a kind of hemolytic disease caused by the immune antibody IgG of fetal red blood cells entering the fetal blood circulation through the placenta, which can cause the destruction of fetal red blood cells. | Internal Medicine-7th. People's Health Publishing House. Beijing, China | 2012 |
| Coagulopathy | Coagulation dysfunction is a hemorrhagic disease caused by coagulation factor deficiency or dysfunction. | Internal Medicine-7th. People's Health Publishing House. Beijing, China | 2012 |
