# Supplementary material for: Differential effects of different delivery methods on progression to severe postpartum hemorrhage between Chinese nulliparous and multiparous women: a retrospective cohort study
Source: BMC Pregnancy Childbirth. 2020 Oct 31;20:660. doi: 10.1186/s12884-020-03351-7 (PMC7603680; doi:10.1186/s12884-020-03351-7)
Supplement: Supplementary file 2 — Additional file 2. Supplementary for Neonatal Characteristics Definition in Table 2. [file 12884_2020_3351_MOESM2_ESM.docx]

**Description of the “Supplementary for neonatal characteristics definition in Table 2”**

This supplementary file aims to explain the neonatal variables of Table 2 in the text of our manuscript. It exhibits the neonatal characteristics variables’ definition and their origins. In this supplementary file, each neonatal variables’ definition is listed in the table in detail. These latest neonatal variables’ concepts are from Chinese textbooks, which are widely applied in neonatal practice in hospitals. Based on this definition, all statistical data related to neonatal diagnosis and treatment are collected. The purpose of this supplementary is to help our readers from different countries to understand the neonatal variables and our research results more easily and clearly.

| Supplementary for Neonatal Characteristics Definition in Table2 | | | |
| --- | --- | --- | --- |
| **Characteristics** | **Definition or Content** | **Origin** | **Year** |
| Vertex malposition | Head position abnormality refers only to other abnormalities of fetal orientation with presentation of fetal head in addition to anterior occipital position. | Obstetrics and Gynecology-8th. People's Health Publishing House. Beijing, China | 2014 |
| Twins or multiplets pregnancy | Twins or Multiples pregnancy is called multiple pregnancies when there are two or more fetuses in the uterine cavity of a single pregnancy. | Obstetrics and Gynecology-8th. People's Health Publishing House. Beijing, China | 2014 |
| Placenta previa | After 28 weeks of gestation, the placenta is located lower than the prenatal part. The placenta previa is attached to the lower part of the uterus and the lower edge of the uterus to reach or cover the inner mouth of the cervix. | Obstetrics and Gynecology-8th. People's Health Publishing House. Beijing, China | 2014 |
| Placenta accreta | Placenta accreta refers to a group of diseases in which placental tissue invades the uterine myometrium to varying degrees | Obstetrics and Gynecology-8th. People's Health Publishing House. Beijing, China | 2014 |
| Placental abruption | Placenta Abruption refers to the normal placenta after 20 weeks of gestation that is partially or completely removed from the uterine wall before the birth of the fetus. | Obstetrics and Gynecology-8th. People's Health Publishing House. Beijing, China | 2014 |
| Placental retention | Placental retention is defined as the absence of a fetus for more than 30 minutes after delivery. | Obstetrics and Gynecology-8th. People's Health Publishing House. Beijing, China | 2014 |
| Amniotic fluid volume abnormality (Polyhydramnios) | During pregnancy, the amount of amniotic fluid exceeds 2000 ml, which is called excessive amniotic fluid. | Obstetrics and Gynecology-8th. People's Health Publishing House. Beijing, China | 2014 |
| Amniotic fluid volume abnormality (Oligohydramnios) | Hypoamniotic fluid is called oligohydramnios when the amount of amniotic fluid in the third trimester of pregnancy is less than 300 ml. | Obstetrics and Gynecology-8th. People's Health Publishing House. Beijing, China | 2014 |
| Premature rupture of membranes(PROM) | Prenatal rupture of membranes is called premature rupture of membranes. | Obstetrics and Gynecology-8th. People's Health Publishing House. Beijing, China | 2014 |
