# Supplementary material for: Differential effects of different delivery methods on progression to severe postpartum hemorrhage between Chinese nulliparous and multiparous women: a retrospective cohort study
Source: BMC Pregnancy Childbirth. 2020 Oct 31;20:660. doi: 10.1186/s12884-020-03351-7 (PMC7603680; doi:10.1186/s12884-020-03351-7)
Supplement: Supplementary file 3 — Additional file 3. Description of the “Supplementary for maternal characteristics definition in Table 1” and Description of the “Supplementary for Neonatal Characteristics Definition in Table 2”. [file 12884_2020_3351_MOESM3_ESM.docx]

**Description of the “Supplementary for maternal characteristics definition in Table 1”**

This supplementary file helps to explain the contents of Table 1 in the text of the manuscript. It exhibits the maternal characteristics variables’ definition and their origins. In this supplementary file, each obstetric definition of maternal characteristics is listed in the table in detail. These latest maternal concepts are from Chinese textbooks, which are nation-widely used in obstetrics practice in hospitals. Based on the standard definition, all statistical data related to obstetric diagnosis and treatment are collected. The purpose of this supplementary is to better enable our readers from different countries to understand the differences of the concepts and the details of obstetrics in China according to the standard. Additionally, it can make sure the readers understand the results of our research more objectively and comprehensively.

**Description of the “Supplementary for neonatal characteristics definition in Table 2”**

This supplementary file aims to explain the neonatal variables of Table 2 in the text of our manuscript. It exhibits the neonatal characteristics variables’ definition and their origins. In this supplementary file, each neonatal variables’ definition is listed in the table in detail. These latest neonatal variables’ concepts are from Chinese textbooks, which are widely applied in neonatal practice in hospitals. Based on this definition, all statistical data related to neonatal diagnosis and treatment are collected. The purpose of this supplementary is to help our readers from different countries to understand the neonatal variables and our research results more easily and clearly.
